# Elixirs of Calisaya and Calisaya and Iron

**Published:** 1868-10-01

**Authors:** X. Toner

**Affiliations:** Albany, New York


### ELIXIRS OF CALISAYA AND CALISAYA AND IRON.

BY X. TONER, M.D., ALBANY, NEW YORK.

THE Cinchonia barks are obtained from trees, or tall shrubs, which are found only in South America, in the higher regions of the Andes. The Cinchonas belong to the natural order *Cinchonaceæ* and to *Pentandria Monogynia* of Linnæus. Their virtues are reported to have been discovered by the Indians, about the year 1500. The story is, that an Indian, while sick of the fever, drank of the waters of a lake in Peru, and soon recovered; and that subsequently so many were cured by the same means when afflicted similarly, that the water came to be regarded in the light of a fever panacea. On investigation, it was found the water owed its healing powers to the bark of these trees, which had been torn up in great abundance by an earthquake, and fallen into it.

Various appellations, from time to time, have been assigned to the Cinchona tribe, in honor of the individuals who introduced it into their respective countries, or sold it as a secret nostrum. Introduced into Europe by the Jesuits it was called *Jesuits' Bark, or Powder;* brought to Rome by a Cardinal, after him it received the epithet *Cardinal del Lugo's Powder;* while in France it might have been heard of as *Talbor's Powders* and *English Remedy*, on account of its eminently successful employment as a nostrum in the hands of Sir Robert Talbor. But the systematic designation Cinchona was applied to the genus of trees producing it by Linnæus, in 1742, in honor of the Spanish Viceroy's lady, the Countess de Chinchon, who was cured of fever by it in Lima, about 1638. She was among the first to test its febrifuge virtues.

The officinal Peruvian barks are *Cinchona flava*, *Cinchona palladi*, and *Cinchona rubra*. Their proximate principles or constituents are numerous, but the most important are *quinia*, *cinchonia*, *quinoidia*, and *quinidia*, which exist in combination with kinic and red cincho-tanic acids.

The yellow bark is very bitter, almost free from astringency, and is comparatively rich in quinia. According to analysis (Waring), one hundred grains of the crude material should yield not less than two grains of this alkaloid. The chief active principle of the pale bark is cinchonine. Two hundred grains should yield not less than two grains of alkaloids. The red bark possesses quinine and cinchonine in about equal proportions; one hundred grains should yield not less than two grains of alkaloids.

All the varieties possess tonic, astringent, and antiperiodic properties, and are of all medicines of their class, the most powerful and uniform in their action. Their medicinal utility depends upon the proportion in which the alkaloids are present in them. As the calisaya cinchona contains the greatest quantity of quinia, and the least amount of the astringent principle, this species receives the most favor with physicians as an antiperiodic and tonic.

The action or mode of action of the calisaya is similar to the action on the animal system of either of the other varieties, whether in its tonic, antiperiodic or sedative action. Its action upon the nervous system is often evinced by a sense of tension, of fullness, or slight pain in the head, or singing in the head, which are always experienced by many individuals, when brought completely under its influence. In the system generally it acts in some way which we don't fully understand, correcting those influences which are probably at work during the respective stages of intermission. In the stomach, the bark excites in a short time warmth in the epigastrium, which in some becomes communicated to the neighboring parts.

Though possessing a three-fold power, bark owes its world-wide reputation to its antiperiodic property; yet it is not employed in the "intermittents" alone. It may be used with benefit in all morbid conditions of the system, whatever may be the peculiar modifications, in which a permanent corroborant effect is desirable, provided the stomach be in a proper state for its reception.

The physician, contending as he frequently has to do, in the peculiar class of cases to which this drug adapts itself, with stomachs easily revolted by the grosser and bulkier medicines, meets with insurmountable objections in the crude material; objections, too, which have thrown many a valuable instrument to combat disease into disuse, practically. 1st. The bark is intensely bitter. This property renders it, *per se*, obnoxious to most patients. 2nd. The dose required to be given in order to produce the desired effect is *enormous*, giving rise, sometimes, to painful and intractable disturbances of the digestive apparatus. As a tonic, the advised dose is from *thirty* to *sixty* grains; as an antiperiodic, the quantity, necessary to prevent the return of the paroxysms, varies from *one* to *eight* drachms, according to whether the attack is simple or pernicious, whether the patient is treated in or at a distance from a miasmatic locality.

These objections have led the profession, through Pharmaceutists, to reduce it to a concentrated and palatable remedy, which comes to us from the manipulations and processes of the laboratory in the form of an elixir. The *Elixir of Calisaya*, as prepared by Tilden & Co., Pharmaceutists, New Lebanon, N. Y., is certainly an elegant medicine, a tonic of paramount excellence. So far as our observation extends, it has the confidence of the profession, and deservedly so. In all the diseases and complaints in which the crude calisaya is beneficial, the elixir proves an agreeable tonic, invigorating the system, improving the appetite, and restoring strength to the weak and debilitated. In low or typhoid forms of disease, in which no inflammation exists, or that which does exist has been moderated by proper remedies, or passed into the suppurative stage, this drug is often of the greatest advantage in supporting the system till the morbid action ceases. Hence its use in the latter stages of typhus gravior, in malignant scarlatina, measles, small-pox; in carbuncles and gangrenous erysipelas, and in all cases in which the system is exhausted under large purulent discharges, and the tendency of the affection is toward recovery. As a tonic there is hardly a disease or complaint connected with debility in which this elixir may not be employed with advantage. No better tonic is recommended in scrofula, dropsy, in obstinate cutaneous affections, chorea, and certain forms of dyspepsia — invigorating the system, improving the appetite, and restoring strength to the weak and debilitated. The strength of the preparation made by Tilden & Co., contains forty grains of the bark to the ounce. Were we called upon to recommend a vegetable tonic, we should unhesitatingly commend to all physicians the *Elixir of Calisaya* prepared by the above named pharmaceutists. We have used it in our own practice with uniformly gratifying results, having never known it to disappoint.

The *Elixir of Calisaya* and *Iron* is another eminently excellent tonic. It is prepared by the same house, and contains in every fluid ounce thirty grains of calisaya and twelve grains of iron. The iron which enters into this preparation is the hypophosphate, acceptable to the most delicate stomach, easily assimilated, and has no tendency to pervert the intestinal secretions, whtch is the result of most chalybeates. It is particularly beneficial in nearly every case of debility partaking of the anæmic condition, in leucocythemia when there is a deficiency of hæmatine in the blood corpuscles. In many cases of defective osseous formation and nervous degeneration, no more available adjuvant has been suggested.

Words seem too tame to adequately portray the advantages these two valuable preparations possess over the crude materials. We are confident when once the physician uses these elixirs, he will never think his medical Armentarium complete without them.

